# Deep Sequencing of the Small RNAs Derived from Two Symptomatic Variants of a Chloroplastic Viroid: Implications for Their Genesis and for Pathogenesis

**DOI:** 10.1371/journal.pone.0007539

**Published:** 2009-10-21

**Authors:** Francesco Di Serio, Andreas Gisel, Beatriz Navarro, Sonia Delgado, Ángel-Emilio Martínez de Alba, Giacinto Donvito, Ricardo Flores

**Affiliations:** 1 Istituto di Virologia Vegetale del CNR, Bari, Italy; 2 Istituto di Tecnologie Biomediche del CNR, Bari, Italy; 3 Instituto de Biología Molecular y Celular de Plantas (UPV-CSIC), Valencia, Spain; 4 Istituto Nazionale di Fisica Nucleare, Bari, Italy; Yale University, United States of America

## Abstract

Northern-blot hybridization and low-scale sequencing have revealed that plants infected by viroids, non-protein-coding RNA replicons, accumulate 21–24 nt viroid-derived small RNAs (vd-sRNAs) similar to the small interfering RNAs, the hallmarks of RNA silencing. These results strongly support that viroids are elicitors and targets of the RNA silencing machinery of their hosts. Low-scale sequencing, however, retrieves partial datasets and may lead to biased interpretations. To overcome this restraint we have examined by deep sequencing (Solexa-Illumina) and computational approaches the vd-sRNAs accumulating in GF-305 peach seedlings infected by two molecular variants of *Peach latent mosaic viroid* (PLMVd) inciting peach calico (albinism) and peach mosaic. Our results show in both samples multiple PLMVd-sRNAs, with prevalent 21-nt (+) and (−) RNAs presenting a biased distribution of their 5′ nucleotide, and adopting a hotspot profile along the genomic (+) and (−) RNAs. Dicer-like 4 and 2 (DCL4 and DCL2, respectively), which act hierarchically in antiviral defense, likely also mediate the genesis of the 21- and 22-nt PLMVd-sRNAs. More specifically, because PLMVd replicates in plastids wherein RNA silencing has not been reported, DCL4 and DCL2 should dice the PLMVd genomic RNAs during their cytoplasmic movement or the PLMVd-dsRNAs generated by a cytoplasmic RNA-dependent RNA polymerase (RDR), like RDR6, acting in concert with DCL4 processing. Furthermore, given that vd-sRNAs derived from the 12–14-nt insertion containing the pathogenicity determinant of peach calico are underrepresented, it is unlikely that symptoms may result from the accidental targeting of host mRNAs by vd-sRNAs from this determinant guiding the RNA silencing machinery.

## Introduction

Viroids are subviral replicons exclusively composed of a small (246–401 nt) circular RNA that, even though lacking protein-coding capacity, is able to infect higher plants and often induce specific diseases [see for reviews 1]–[5]. Viroids are classified into two families: *Pospiviroidae*, the members of which replicate in the nucleus through an asymmetric rolling-circle mechanism catalyzed by host enzymes [6], [7], and *Avsunviroidae*, whose members replicate in the chloroplast through a symmetric rolling-circle mechanism involving host enzymes and *cis-*acting hammerhead ribozymes embedded in the viroid strands of both polarities [8], [9].

Despite their fundamental differences with viruses in structure, function and evolutionary origin, research on viroids has been deeply influenced by previous discoveries on viruses and, more specifically, on plant riboviruses. This is particularly the case of pathogenesis —symptoms incited by viruses and viroids are to a good extent similar— suggesting that some steps of the underlying mechanism might be shared by both biological entities. Pertinent to this context is the finding that higher eukaryots, including plants, have evolved an RNA-based antiviral silencing response [see for reviews 10], [11], to which viruses have reacted with counter-defense mechanisms by encoding in their genomes suppressors of RNA silencing [see for reviews 12], [13]. Furthermore, because RNA silencing not only plays an antiviral role but it is also involved in regulating plant development, with both routes being partially overlapping [see for reviews 14], [15], viral symptoms resembling developmental defects have been regarded as a side effect of virus suppressors acting concurrently on the two routes [16]. Other data, however, indicate that symptoms induced by plant viruses are not necessarily a direct consequence of their suppressors [see for a review 13]. Altogether these results raise the question of whether viroids are also triggers, targets and even suppressors of the RNA silencing machinery of their hosts.

Northern-blot hybridization of nucleic acid preparations from plants infected by distinct members of the two viroid families have revealed 21–24-nt viroid-derived small RNAs (vd-sRNAs) with the characteristic properties of the small interfering RNAs (siRNAs) generated by Dicer-like RNases (DCLs) [17], [18], the RNAs guiding the sequence-specific step of RNA silencing, strongly supporting that this machinery is activated by viroids [19]–[22]. Moreover, low-scale sequencing of the vd-sRNAs from two members of the family *Pospiviroidae* [23]–[25] and very recently of one of the *Avsunviroidae* [26] has confirmed that they are predominantly of 21 and 22 nt and (+) polarity, although there are some discrepancies even between results derived from the same viroid-host combination —with one report describing an abundant cluster of (−) sRNAs [24]— that may be in part the consequence of the limited dataset generated by low-scale sequencing.

*Peach latent mosaic viroid* (PLMVd) [27], is the type species of the genus *Pelamoviroid* within the family *Avsunviroidae* [28], [29]. Infections by PLMVd often occur without eliciting visible foliar symptoms, although some isolates incite a typical peach mosaic (PM) and others peach calico (PC), an extreme chlorosis (albinism) that may cover the complete leaf lamina [see for a review 30]. Cloning and sequencing have revealed that PLMVd isolates are formed by complex populations of sequence variants [31], and bioassays in the peach indicator GF-305 with *in vitro* transcripts of individual cDNA clones have shown that genetic variability is rapidly restored as a result of the highly error-prone replication [32], [33]. These studies have identified three classes with specific biological properties: latent and PM-inducing variants of 335–338 nt (31,32), and PC-inducing variants of 348–351 nt with a 12–14-nt insertion that contains the pathogenicity determinant [34]–[36]. The pathogenicity determinant of PM, however, remains unknown.

Here we report the first deep sequencing applied to vd-sRNAs, specifically to the PLMVd-sRNAs accumulating in peach leaves infected by two molecular variants inducing PM and PC, and discuss the implications for the genesis of the vd-sRNAs and for viroid pathogenesis.

## Materials and Methods

### Sources of PLMV-infected tissue

Young leaves were collected from greenhouse-grown seedlings of GF-305 peach (*Prunus persica*, Bastch) slash-inoculated with *in vitro* transcripts of two natural PLMVd variants: PC-C40 [34]–[36], which induces PC (albinism) and GDS6 [31], [32] inciting PM (a typical mosaic). In PC-expressing leaves the albino area covered essentially the whole blade, while in PM-expressing leaves the chlorotic and the adjacent green areas were interspersed.

### Extraction and fractionation of peach sRNAs

Total nucleic acids were extracted from leaf pieces with phenol-chloroform-isoamilic alcohol and recovered from the aqueous phase by ethanol precipitation [36]. Nucleic acids were separated by PAGE on 17% gels containing 1XTBE and 8 M urea, with oligodeoxyribonucleotides of known size (18–27 nt) in the external lanes that, after staining with ethidium bromide, serve to delimit the gel section where the sRNAs had migrated. The sRNAs were susbsequently eluted, recovered by ethanol precipitation and quantitated with a spectrophotometer (Nanodrop).

### Amplification and sequencing of peach sRNAs

The sRNAs were subjected to: 1) single-stranded ligation of bar-coded 5′ adapter, 2) PAGE purification, 3) single-stranded ligation of 3′ adapter, 4) PAGE purification, 5) reverse transcription and PCR amplification to generate the DNA colony template library, 6) PAGE purification, 7) Quality control of the DNA colony template library by cloning an aliquot into a TOPO plasmid and capillary sequencing of 8–10 clones, 8) library purification, estimation of the concentration and dilution at 10 nM, 9) Flow-cell preparation on the cluster station, and 10) high-throughput DNA sequencing on the Illumina Genome Analyzer EAS269-GAII (FASTERIS SA, Plan-les-Ouates, Switzerland). The two bar-coded samples (PC-C40 and GDS6) were analyzed in a single read channel.

### Sequence analysis of PLMVd-sRNAs

The resulting sequences were examined for the presence of the adapters and, after their trimming, they were sorted into separate files according to their length. For further analysis the sequences between 20 and 24 nt were pooled and each set of sequences was analyzed by BLAST [37] against the nucleotide sequence of the inoculated PC-C40 and GDS6 variants and their known progenies. No mismatch was allowed and the circularity of the viroid genome was taken into consideration. A set of perl scripts to filter, analyze and visualize the mapping data, serching for specific distribution patterns and phasing was developed.

### Northern-blot hybridization of PLMVd-sRNAs with oligodeoxyribonucleotides

Aliquots of the same total nucleic acid preparation from PC-expressing leaves used in deep sequencing were fractionated by PAGE as indicated above, electrotransferred and fixed by UV irradiation to nylon membranes (Hybond-N, Amersham). The membranes were hybridized at 37°C in Perfect-Hyb buffer (Sigma) with each of the following 5′-radiolabeled probes: PL-1 (5′-GTTCCCGAAGGAAAAGTCCCACCTTACCTCATTGCG-3′) and PL-2 (5′- GGTGGAGGGGCTGAGAGGTCGCTACTCTCTCAAAAG-3′) complementary to positions 138–173 and 51–86, respectively, and PL-3 (5′- GAGAGAGTGGCGACCTCTCAGCCCCTCCACCTTGGGG-3′) and PL-4 (5′- GAGTCTCTGAAATGAGACGAAACTCTTCAAGAACTTTTGTTCC-3′) identical to positions 56–92 and 308-1, respectively, of the PC-C40 variant. Some minor changes with respect to the PC-C40 sequence were introduced in the probes for taking into account the variability observed in the sequenced PLMVd-sRNAs. After overnight hybridization, the membranes were washed twice with 2X SSC plus 0.1% SDS for 10 min at room temperature, and once with 0.1X SSC plus 0.1% SDS at 55°C for 15 min, and examined with a bioimage analyzer (Fujifilm FLA-5100).

## Results

### Multiple PLMVd-sRNAs from peach infected by two symptomatic molecular variants

**Figure 1:**
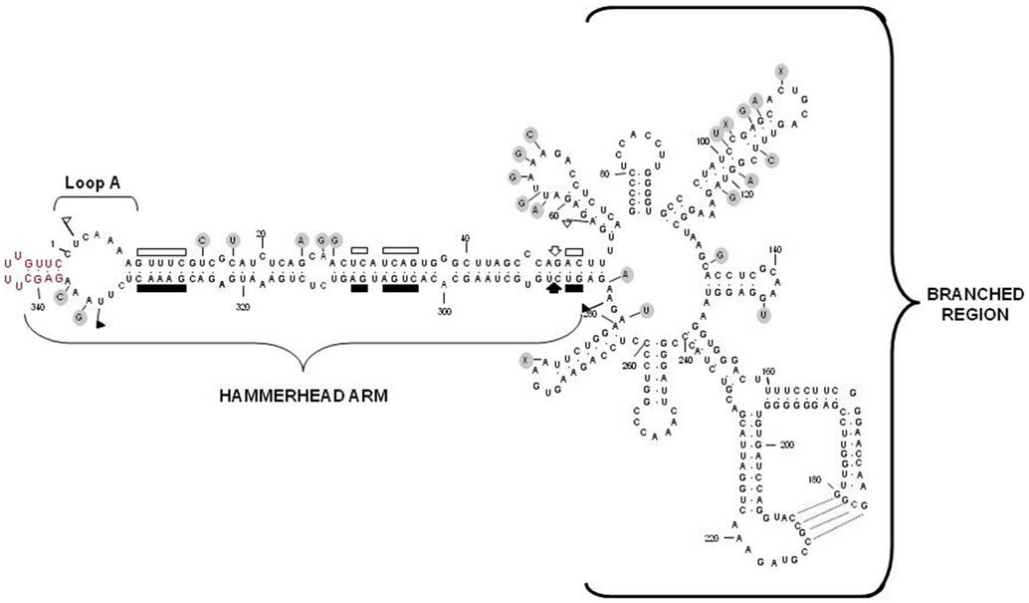
Sequence and predicted secondary structure for the (+) strand of the PLMVd variant PC-C40. Residues forming the insertion in loop A containing the PC determinant are highlighted in red [34], [36]. Self-cleaving (+) and (−) domains are delimited by flags, residues conserved in most natural hammerhead structures are denoted by bars, and self-cleavage sites are marked by arrows. Closed and open symbols refer to (+) and (−) polarities, respectively. Residues involved in a pseudoknot between positions 177 to 180 and 210 to 213, proposed on the basis of *in vitro* assays [70] and *in vitro* and *in vivo* data obtained with another viroid of the same genus [71], are indicated by broken lines. The reference PLMVd variant GDS6 inducing PM lacks the insertion in loop A and presents additional changes spread throughout the molecule (denoted with grey circles) that preserve the branched conformation [31]. Note that the left part of the proposed secondary structure (the so-called hammerhead arm) forms a rod-like folding while the right part adopts a branched conformation with multiple hairpins. The computer-predicted secondary structure for the PLMVd (−) strand also contains the hammerhead arm and a branched region with multiple hairpins (data not shown).

We carried out our deep sequencing study starting from two preparations of gel-purified sRNAs from GF-305 peach seedlings infected by PC-C40 and GDS6 variants (Fig. 1). Each preparation was linked to a bar-coded adapter in order to sequence the two libraries in the same channel, thus avoiding any undesired bias and obtaining two independent datasets that could be further compared. Moreover, PLMVd-sRNA analysis was simplified because the progenies resulting from the two specific PLMVd variants, which come from greenhouse plants that have been infected for a short time, are considerably less complex populations than those from field trees [26] that may have been infected (and even re-infected) for a long time. Additionally, we had previous information about the sequence of eight variants from the GDS6 progeny [32] and of 16 variants from the PC-C40 progeny (Navarro et al., unpublished data), which was used in the subsequent analysis. Adapters were synthesized assuming that the vd-sRNAs have the characteristic 5′ phosphomonoester and 3′ hydroxyl termini resulting from DCL activity [17], [18]. Although earlier data indicate that this maybe the case [25], [26], further studies are needed to exclude the possibility that a fraction of the vd-sRNAs may have other 5′ termini as recently reported for secondary siRNAs in *Caenorhabditis elegans* [38], [39].

**Figure 2:**
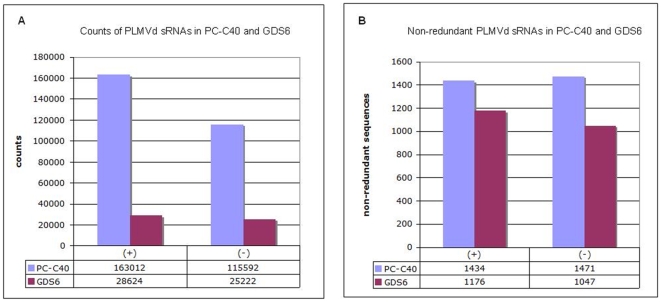
Redundant and non-redundant PLMVd-sRNAs. Histograms comparing the total counts (A) and number (B) of non-redundant (+) and (−) PLMVd-sRNAs (20–24 nt) obtained by deep sequencing of samples PC-C40 and GDS6.

Of about 5490000 reads obtained by the high-troughput sequencing, 97% were clearly attributable to the corresponding bar-coded samples (42% for the PC-C40 and 56% for GDS6 samples, respectively) and adopted a profile with two prominent 21- and 24-nt peaks (excluding the files with inserts without adapters and empty inserts) (Table S1 and Fig. S1). When the inserts between 20 and 24 nt (1048070 for the PC-C40 and 1349816 for GDS6 samples, respectively) were searched for PLMVd-sRNAs matching perfectly the sequence of PC-C40 (and its progeny variants) and of GDS6 (and its progeny variants), they resulted into 278604 and 53846 counts, respectively (Fig. 2A). This 5-fold difference most likely reflected the initial sRNA concentration, which was higher in the PC-C40 preparation than in the GDS6 one as revealed by spectrophotometry and molecular hybridization with a PLMVd-specific probe (data not shown). Highly repetitive counts most likely resulted from the most abundant vd-sRNAs. However, the non-redundant (unique) PLMVd-sRNAs were comparable in the PC-C40 and GDS6 preparations (2905 and 2226, respectively) (Fig. 2B), indicating a similar level of qualitative complexity in both cases.

### 21-nt (+) and (−) RNAs in similar proportion dominate the PLMVd-sRNA profile

**Figure 3:**
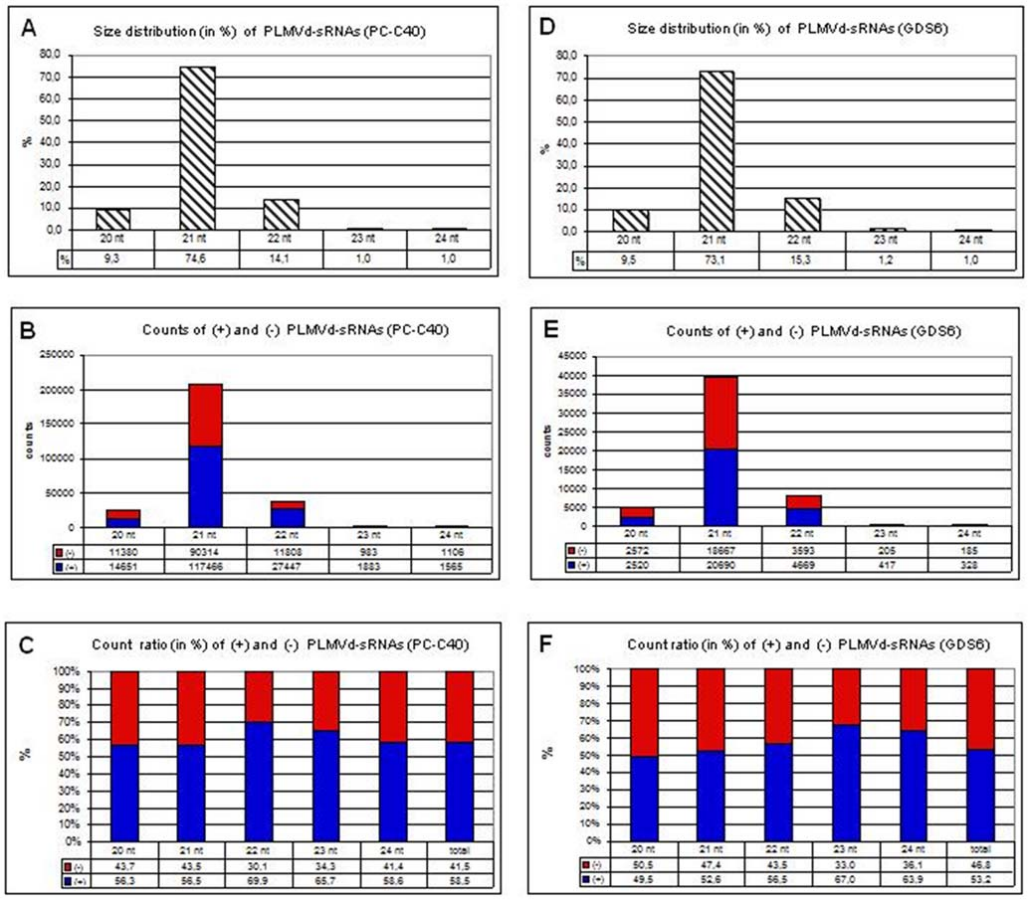
Size and abundance of the PLMVd-sRNAs. Histograms comparing in samples PC-C40 (upper panels) and GDS6 (lower panels) the size distribution of 20–24 nt PLMVd-sRNA counts (A and D), the total counts of (+) and (−) PLMVd-sRNAs (B and E), and the count ratio of (+) and (−) PLMVd-sRNAs (C and F).

Analysis of the size distribution of the counts corresponding to the PC-C40 sample revealed that about 75% were of 21 nt, 15% of 22 nt and 10% of 20 nt, while those of 23 and 24 nt represented only 1% each (Fig. 3A). Interestingly, the size distribution of the counts corresponding to the GDS6 sample was very similar (Fig. 3D), supporting the reproducibility of the sequencing approach and that, in global terms, similar DCLs seem to act on PLMVd-specific RNA substrates in tissues displaying very different symptoms (PC and PM). Moreover, the asymmetrical size distribution of the PLMVd-sRNA profile suggests that the DCLs involved in their genesis operate hierarchically (see below).

Regarding polarity, approximately 60 and 40% of the PC-C40 counts were of (+) and (−) polarity, respectively, with a similar ratio being observed for the counts corresponding to 20-, 21- and 24-nt PLMVd-sRNAs, whereas in those corresponding to 22- and 23-nt PLMVd-sRNAs the fraction of (+) polarity was higher (around 70%) (Fig. 3B and C). Data derived from the GDS6 sample were similar: the balance between the polarities of the GDS6 counts was close to 50% in total counts and in those corresponding to 20-, 21- and 22-nt PLMVd-sRNAs, with the (+) polarity being predominant (around 65%) in those corresponding to 23- and 24-nt PLMVd-sRNAs (Fig. 3E and F). The similar proportion or the slightly surplus of the (+) PLMVd-sRNAs parallels the ratio between the genomic RNAs of both polarities in infected tissue, wherein there is also a slight excess of circular and linear (+) monomeric forms with respect to their complementary counterparts [36], [40], [41]. Although this parallelism suggests a direct precursor-product relationship between the PLMVd genomic RNAs and the vd-sRNAs, this must not be necessarily the case (see below).

### A biased distribution of the 5′ nucleotide in (+) and (−) PLMVd-sRNAs

**Figure 4:**
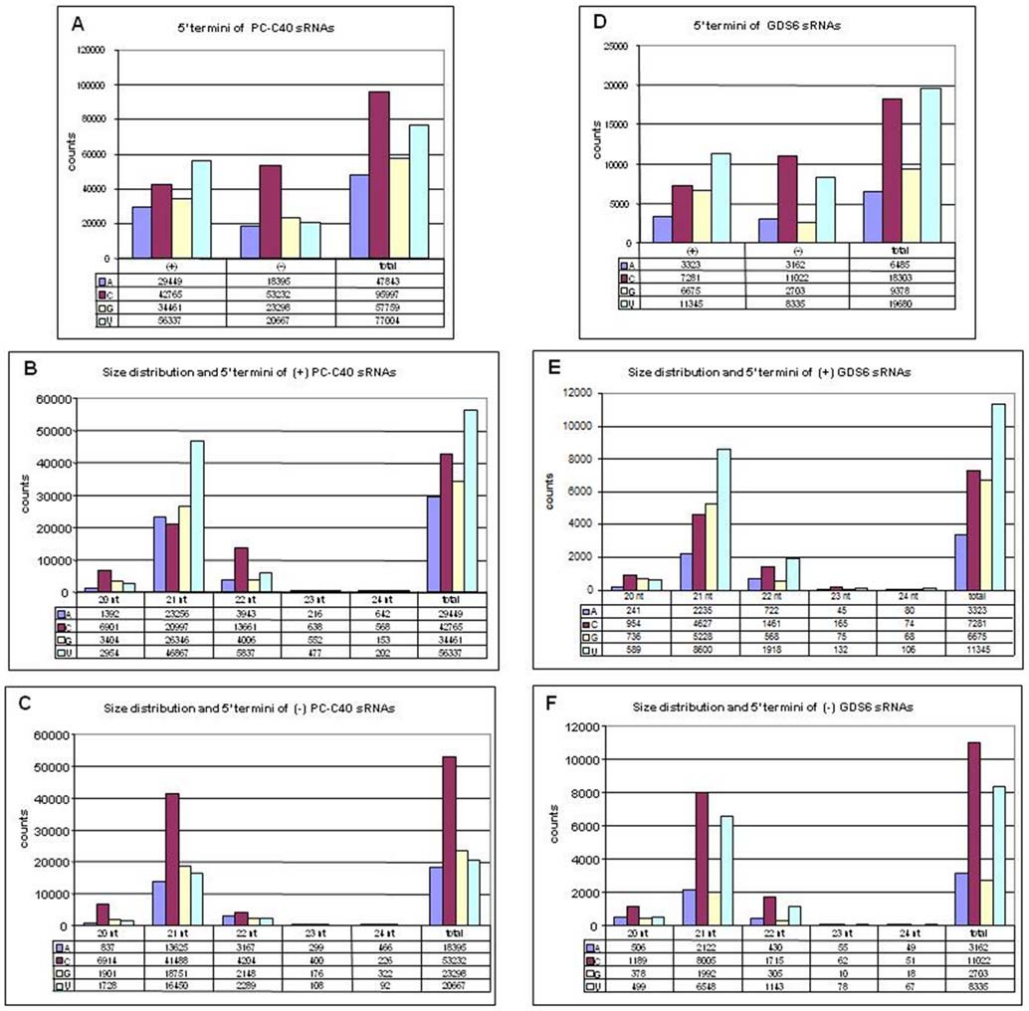
Relative abundance of PLMVd-sRNAs with distinct size and 5′ termini. Histograms comparing in samples PC-C40 (upper panels) and GDS6 (lower panels) the total counts corresponding to (+) and (−) PLMVd-sRNAs (20–24 nt) with different 5′ termini (A and D), and the size distribution and 5′ termini of (+) PLMVd-sRNA counts (B and E) and of (−) PLMVd-sRNA counts (C and F).

Analysis of the 5′-terminal positions in the PC-C40 sample revealed the prevalence of C and U residues, with U dominating in the (+) PLMVd-sRNAs and C in their (−) counterparts (Fig. 4A). A similar distribution was observed for the 21-nt RNAs as anticipated from their preponderance within the PLMVd-sRNAs, while C was the most frequent 5′ nucleotide in the 22-nt (+) RNAs and only minor differences were observed within the 22-nt (−) RNAs (Fig. 4B and C). A parallel analysis of the 5′-terminal positions in the GDS6 sample produced similar results except that U was the most frequent nucleotide in the 22-nt (+) RNAs, reinforcing again the reproducibility of the deep sequencing approach. Leaving apart the redundancy of the specific 5′ termini and their allocation along the genomic (+) and (−) RNAs, which is described in the next section, these data by themselves are indicative of an uneven distribution because PLMVd is rich in G+C [27]. This distribution may result from the combined action of several DCLs and the subsequent loading of the vd-sRNAs into the final effectors of RNA silencing, the core of which bind the 5′ termini of their guide sRNAs with different affinity (see below). Moreover, the possibility exists that PLMVd-sRNAs might be differentially targeted by one or more exoribonucleases of the SMALL RNA DEGRADING NUCLEASE (SDN) family that act upon mature miRNAs in Arabidopsis [42].

### PLMVd-sRNAs map along the genomic (+) and (−) RNAs adopting a hotspot profile

**Figure 5:**
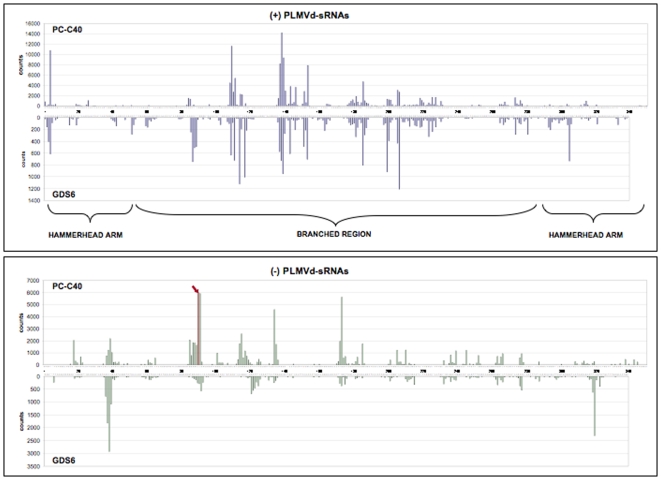
Location and frequency of the 5′ termini of the PLMVd-sRNAs in the primary structure of the genomic RNAs. Note that the scale of counts is different in both samples, that only frequencies above 50 and 10 for the PC-C40 and GDS6, respectively, are included, and that the same numbers are used in the (+) polarity (5′→3′ orientation is from left to right) and in the (−) polarity (5′→3′ orientation is from right to left). The arrow on top of the red bar indicates that the counts (32260) of (−) PLMVd-sRNAs with 5′ terminus at position 90 are out of the scale. For the location and frequency of the 5′ termini of the PLMVd-sRNA we have considered the parental infecting variants PC-C40 and GDS6 and their known progenies. The genomic PLMVd RNAs of PC-C40 and GDS6 variants are presented as their corresponding DNAs.

**Figure 6:**
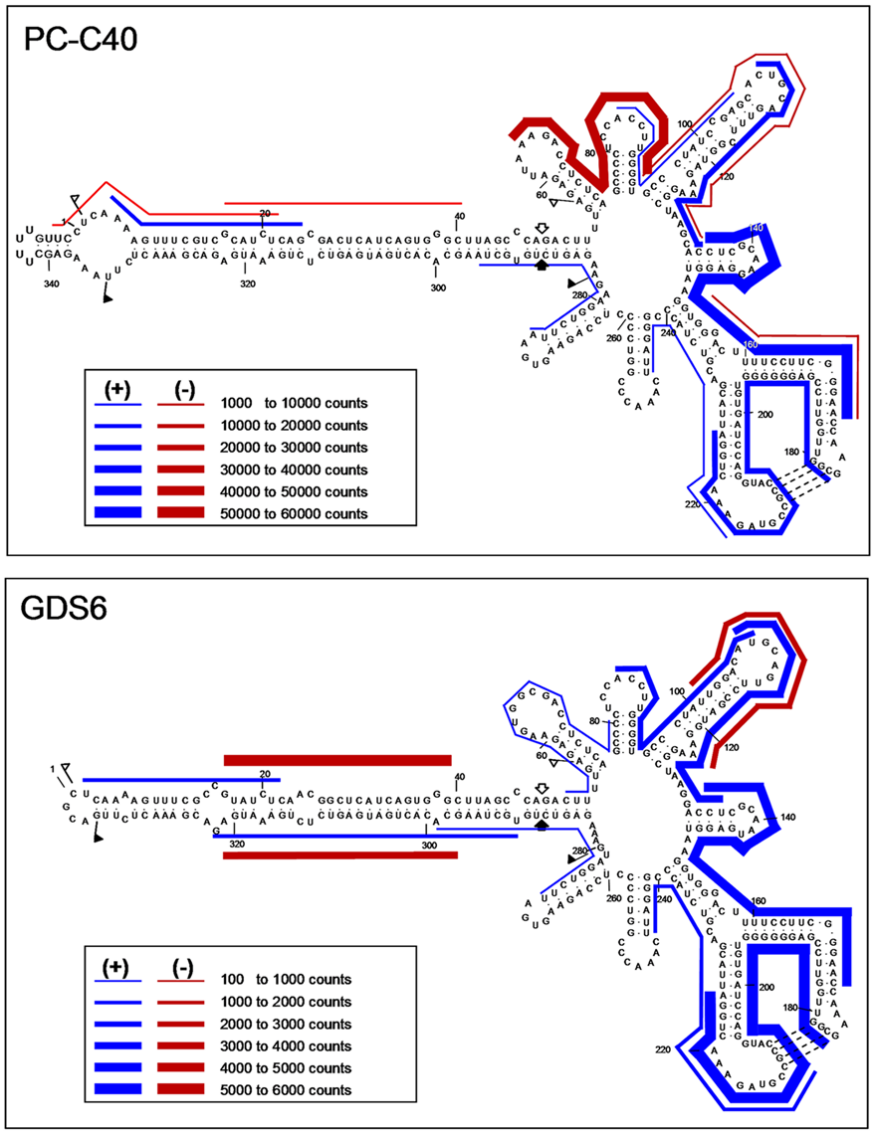
Location and frequency of the 5′ termini of the PLMVd-sRNAs in the secondary structure of the genomic PLMVd (+) RNA. Note that the scale of counts is different in both samples and that only frequencies above 1000 and 100 for the PC-C40 and GDS6, respectively, are included. The PLMVd-sRNAs of both polarities are referred to the secondary structure of the genomic PLMVd (+) RNA, which is based in thermodynamic, *in vitro*, and *in vivo* data.

Analysis of the PLMVd-sRNA frequency revealed that essentially all positions of the genomic (+) and (−) RNAs were represented in the 5′-termini of the PLMVd-sRNAs; however, their distribution was uneven, with a large fraction of the counts concentrating in specific regions (hotspots). Several aspects are noteworthy. First, the hotspot patterns formed by the (+) PLMVd-sRNAs of the PC-C40 and GDS6 samples are similar (Fig. 5), as well as those formed by the (−) PLMVd-sRNAs of the two samples; however, the hotspot patterns of (+) and (−) PLMVd-sRNAs are different, indicating that DCL processing of their corresponding precursors is a polarity intrinsic property. Second, the most prominent hotspots, with the exception of some (−) vd-sRNAs in the GDS6 sample, did not map at the so-called hammerhead arm (Fig. 1 and 6) –a potential substrate for DCLs because in both genomic (+) and (−) RNAs it folds into a long and almost perfect dsRNA structure according to *in silico* predictions [27], [31] *in vitro* digestions [43] and natural covariations [31], [32]– but rather at the right moiety of the molecule formed by multiple and shorter hairpins (Fig. 6). Therefore, the PLMVd genomic (+) and (−) RNAs by themselves do not account for the production of the observed vd-sRNA profile. Moreover, because the hotspot profile of the 21- and 22-nt PLMVd-sRNAs is very similar (Fig. 5 and data not shown), this profile does not seem imposed by the specific DCLs involved but rather by their RNA substrates. Third, a search identified some perfectly complementary vd-sRNAs with two 3′-protruding nucleotides in each strand, exemplified by the most abundant PLMVd-sRNAs of minus polarity, the 5′ terminus of which is located in the hammerhead arm around positions 40 and 320 in the GDS6 sample (Fig. 6); however they should not be neccessarily regarded as direct DCL products because a control search for perfectly complementary vd-sRNAs with two 5′-protruding nucleotides in each strand produced frequent duplexes without apparent physiological role. Fourth, one specific (−) vd-sRNAs of 21 nt was unusually abundant in the PC-C40 sample (it was also present in the GDS6 sample, but with a considerable lower frequency). This PLMV-sRNA (5′-CCAAGGUGGAGGGGCUGAGAG-3′) is rich in G+C; a bias of this class in *Turnip mosaic virus* siRNAs has been interpreted as result of DCLs targeting preferentially GC-rich regions [44]. Fifth, this unusually abundant PLMVd-sRNA is in a 21-nt phase with the 3′ end resulting from the (−) hammerhead ribozyme; however, we have been unable to detect a consistent phasing in the PLMVd-sRNAs from both samples. And sixth, the (+) and (−) vd-sRNAs derived from the determinant of PC symptomatology (Fig. 5 and 6, positions 338–349) are underrepresented, a result with implications in the mechanism of viroid pathogenesis (see below).

### Molecular hybridization with specific oligodeoxyribonucleotides confirms the relative abundance of some representative PLMVd-sRNAs

**Figure 7:**
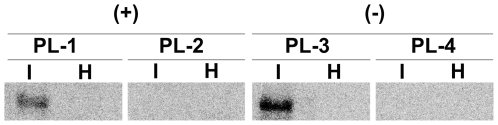
Confirmation by molecular hybridization of the relative abundance of some representative PLMV-sRNAs. Northern-blot hybridizations with 5′-radiolabeled oligonucleotides PL-1 and PL-2 for detecting abundant and scarce (+) PLMVd-sRNAs, respectively, and PL-3 and PL-4 for detecting abundant and scarce (−) PLMVd-sRNAs, respectively. H and I refer to healthy and infected (with the PC-C40 variant) RNA preparations, respectively. After hybridization, the membranes were examined with a bioimage analyzer (Fujifilm FLA-5100).

To obtain independent validation of our data, excluding the possibility that the differences observed in the PLMVd-sRNA profiles could result from amplification artifacts, we estimated by Northern-blot hybridization the relative abundance of some vd-sRNAs derived from regions of the PC-C40 genome wherein deep sequencing mapped high and low density of vd-sRNAs. The hybridization signals generated by probes PL-1 and PL-3, complementary to regions with high density of (+) and (−) vd-sRNAs respectively, were clearly stronger than those generated by probes PL-2 and PL-4, complementary to regions with low density of (+) and (−) vd-sRNAs respectively (Fig. 7). These results, therefore, support again the reliability of the deep sequencing approach.

## Discussion

Infection by viroids of both families elicits the accumulation of 21–24 nt vd-sRNAs (see above) and co-delivery of homologous double-stranded RNA (dsRNAs), mechanically or by agroinoculation, interferes with viroid infection [45]. Together these results support the view that viroids trigger the RNA silencing machinery of their hosts and are targeted by: i) one or more DCLs, which are secondary structure-specific and, ii) one or more Argonautes (AGO) forming the core of the RNA-induced silencing complex (RISC), which are sequence-specific [see for a review 15]. Viroid titer could thus be regulated by the concerted action of DCL and RISC, with DCLs processing the highly-structured genomic viroid RNA or some viroid dsRNAs arising from replication or from the action of host RDRs (in contrast to viruses viroids do not encode their own RNA replicases), and the resultant vd-sRNAs priming RISC and targeting additional viroid RNAs for degradation [2], [45]. The vd-sRNAs may also have a direct role in pathogenesis by acting like microRNAs or trans-actingRNAs and targeting endogenous mRNAs for inactivation [20], [46], [47]. In this scenario, vd-sRNAs harboring the pathogenicity determinants mapped in some viroids, including PLMVd [34], [35], should be well-represented in the vd-sRNA populations.

To focus on the *bona fide* PLMVd-sRNAs present in the two datasets of peach sRNAs here obtained by deep sequencing, we retrieved only those matching perfectly the sequence of the parental variants (PC-C40 and GDS6) and their known progenies. Our results show that the population of PLMVd-sRNAs in both samples is dominated (75%) by 21-nt (+) and (−) RNAs in similar proportion, with the remaining components being (+) and (−) RNAs of 22 nt (15%) and 20 nt (10%), and minimal fractions (1%) of 23- and 24-nt RNAs. These figures are in good agreement with sequencing data of 60 PLMVd-sRNAs from infected field trees [26], although there are major differences in the methodologies and inferences between both studies (see below). In contrast, low-scale sequencing of the vd-sRNAs from two members of the family *Pospiviroidae*, *Potato spindle tuber viroid* (PSTVd) and *Citrus exocortis viroid* (CEVd), has revealed two prominent peaks of 21- and 22-nt (+) RNAs [23], [25].

Previous studies using tomato, the preferred experimental host for PSTVd, have shown that PSTVd replicates in the nucleus and that (+) strands accumulate to significantly higher levels than (−) strands [6]. Assuming that the subcellular localization of tomato DCLs and the size of the sRNAs they generate are the same as those of their Arabidopsis homologues, the most likely candidate for producing the 21-nt (+) PSTVd-sRNAs is DCL1, which processes the nuclear miRNA precursors with a secondary structure resembling that of the genomic PSTVd (+) RNA [48]; the 22-nt (+) PSTVd-RNAs could emerge from an alternative pathway (see below). On the other hand, PLMVd replicates in peach plastids wherein the accumulation of (+) strands exceeds only slightly that of the (−) strands [36], [40], [41]. Extending to peach the properties previously assumed for tomato DCLs, DCL4 and DCL2 —which act hierarchically in antiviral defense [49]–[52]— are the most likely players in the genesis of the 21- and 22-nt PLMVd-sRNAs, respectively, rather than the DCL1 proposed previously [26]. Because RNA silencing has not been reported in plastids so far, DCL4 and DCL2 should dice either the PLMVd genomic RNAs during their cytoplasmic movement [21] or the PLMVd-dsRNAs generated by a cytoplasmic RNA-dependent RNA polymerase (RDR), like RDR6, which acts in concert with DCL4 processing [53]. This same route could account for the production of the 22-nt and part of the 21-nt (+) RNAs of PSTVd. However, it is surprising the low frecuency of 24-nt vd-sRNAs detected by low-scale sequencing in tissues infected by PSTVd and CEVd [23]–[25], which having a nuclear accumulation site appear as good candidates for being also targeted by DCL3. Deep sequencing studies should help to clarify this issue.

The distribution of the most abundant PLMVd-sRNAs along the genomic (+) and (−) RNAs, which has been independently confirmed by molecular hybridization, also supports that they are not the only substrates upon which DCLs operate, because there is not a good correlation between hotspots and regions predicted to adopt a compact secondary structure; some data in this direction were obtained in a previous low-scale sequencing of PLMVd-sRNAs [26], although their distribution was somewhat different. One or more RDR(s) producing PLMVd-dsRNAs are thus presumably involved, which could be activated by the peculiar structural features of the genomic viroid RNAs, circular and linear strands without cap structure and poly-A tail that might be recognized as aberrant RNAs [54], [55]; actually, some hotspots are in a 21-nt phase with the hammerhead-mediated self-cleavage sites in both polarities. Implicit in this proposal is that a significant fraction of the vd-sRNAs are secondary RNAs and that, in contrast with the secondary siRNAs from *C. elegans*, they do not result from unprimed RNA synthesis [38], [39]. In plants, secondary virus-derived sRNAs have been involved in the maintenance step of virus-induced RNA silencing [56], with secondary vd-sRNAs perhaps playing a similar role. Alternative scenarios that may influence the hotspot profile of vd-sRNAs would include shielding of certain domains of the genomic (+) and (−) RNAs by proteins [57], [58], and the differential stability of the vd-sRNA strands as a result of their 3′ methylation mediated by HEN1 [59], [see for a review 60] and incorporation into effectors complexes like RISC containing distinct AGO members. Recent evidence indicates that virus-derived small RNAs act as genuine siRNAs and program RISC for virus RNA degradation [61], [62]. More specifically, AGO1 and AGO7 mediate clearance of *Turnip crinkle virus* —with AGO1 targeting viral RNAs with compact structures and AGO7 and RDR6 less-structured RNAs [52]— while AGO1 mutants are impaired in resistance to *Cucumber mosaic virus* (CMV) [63], and AGO2 and AGO5 have the ability to bind CMV-derived sRNAs [64]. In this context, the 5′-terminal nucleotide of the sRNA plays a key role in the sorting process [see for a review 65]. Direct proof that vd-sRNA indeed target the viroid genomic RNAs, which due to their secondary structure may offer some resistance to RISC [23], [66], and elucidation of the AGO(s) involved, remain intriguing issues. Recent results indicate that transgenic tomato plants expressing a non-infectious PSTVd hairpin-RNA construct exhibit resistance to PSTVd infection; this resistance is correlated with high accumulation of hairpin-derived siRNAs that appear, therefore, to effectively target the mature viroid RNA [67].

Our results also bear some implications for pathogenesis. Because no hotspots were mapped at the 12–14 nt insertion containing the pathogenicity determinant of PC [34]–[36], it is unlikely that the symptoms may result from the accidental targeting of host mRNAs by a vd-sRNAs generated from the determinant as proposed previously [20], [46], [47], [see for a review Barba and Hadidi 68]. In line with this view, low-scale sequencing has also revealed that vd-sRNAs derived from the pathogenicity domain are not particularly abundant in PSTVd- and CEVd-infected tissues [23]–[25] and, in contrast with previous results [46], a recent report shows that the transgenic tomato plants expressing a non-infectious PSTVd hairpin-RNA construct do not display symptoms despite accumulating abundant hairpin-derived siRNAs [67]. Therefore, the genomic RNAs could be the ultimate effector of pathogenesis or even the replication process itself, which may detract components of the RNA silencing machinery and impair its normal functioning. Pertinent to this context is the finding that suppression of RNA silencing in a specific RNA virus can be induced by the viral replication complex [69].

Finally, our deep sequencing study has also retrieved a wide spectrum of endogenous peach sRNAs. A detailed description of the characteristics of these sRNAs and of the effects on their distribution profile caused by the infection with the two phenotypically different PLMVd variants, PC-C40 and GDS6, will be reported elsewhere. Here we would like just to mention that while the 21-nt peach sRNAs dominate in the PC-C40 sample, the 24-nt sRNAs are the most abundant species in the GDS6 sample (Fig. S1).

## Supporting Information

Table S1 Length and counts of sequenced inserts from PC-C40 and GDS6 samples.
(0.07 MB TIF)

Figure S1 Length and counts of sequenced inserts from PC-C40 and GDS6 samples. The fraction (%) of inserts of a given length in each sample has been plotted against the given length.
(0.07 MB TIF)
