# Supplementary figures and images for: Deep Sequencing of the Small RNAs Derived from Two Symptomatic Variants of a Chloroplastic Viroid: Implications for Their Genesis and for Pathogenesis
Source: PLoS One. 2009 Oct 21;4(10):e7539. doi: 10.1371/journal.pone.0007539 (PMC2760764; doi:10.1371/journal.pone.0007539)

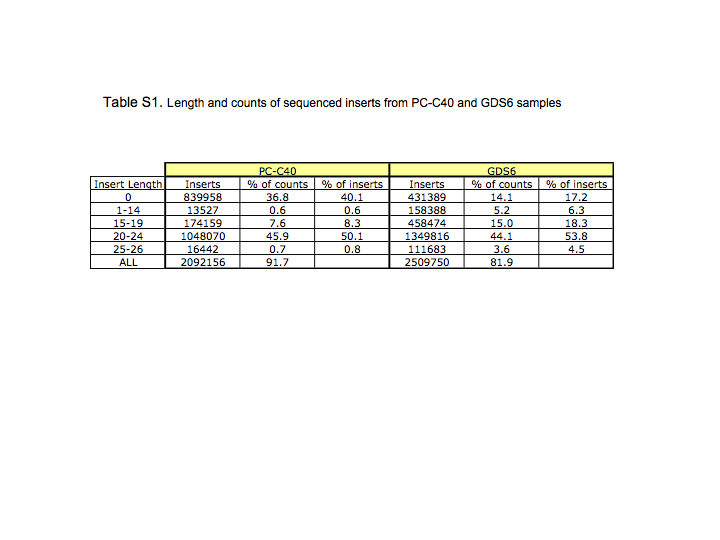

Supplement: Table S1 — Length and counts of sequenced inserts from PC-C40 and GDS6 samples. (0.07 MB TIF) [file pone.0007539.s001.tif]

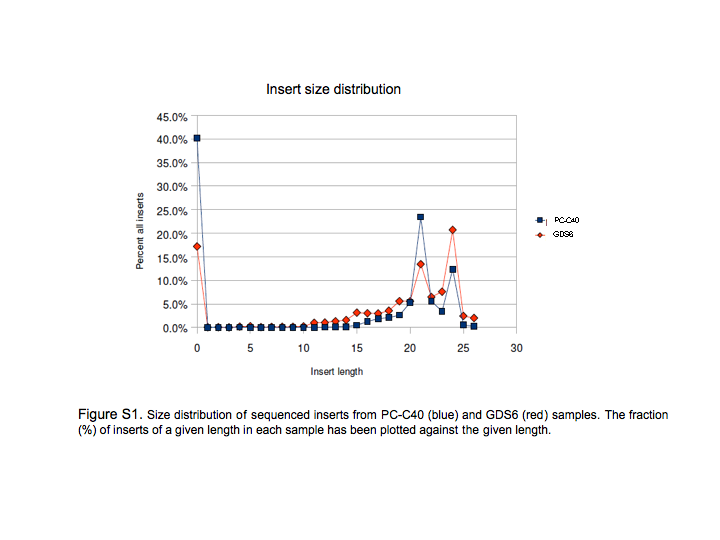

Supplement: Figure S1 — Length and counts of sequenced inserts from PC-C40 and GDS6 samples. The fraction (%) of inserts of a given length in each sample has been plotted against the given length. (0.07 MB TIF) [file pone.0007539.s002.tif]
